# Echocardiography as a Tool to Assess Cardiac Function in Critical Care—A Review

**DOI:** 10.3390/diagnostics13050839

**Published:** 2023-02-22

**Authors:** Marius Keller, Harry Magunia, Peter Rosenberger, Michael Koeppen

**Affiliations:** Department of Anesthesiology and Intensive Care Medicine, University Hospital Tübingen, Hoppe-Seyler-Straße 3, 72076 Tübingen, Germany

**Keywords:** echocardiography, critical care, hemodynamics

## Abstract

In critically ill patients, hemodynamic disturbances are common and often lead to a detrimental outcome. Frequently, invasive hemodynamic monitoring is required for patients who are hemodynamically unstable. Although the pulmonary artery catheter enables a comprehensive assessment of the hemodynamic profile, this technique carries a substantial inherent risk of complications. Other less invasive techniques do not offer a full range of results to guide detailed hemodynamic therapies. An alternative with a lower risk profile is transthoracic echocardiography (TTE) or transesophageal echocardiography (TEE). After training, intensivists can obtain similar parameters on the hemodynamic profile using echocardiography, such as stroke volume and ejection fraction of the right and left ventricles, an estimate of the pulmonary artery wedge pressure, and cardiac output. Here, we will review individual echocardiography techniques that will help the intensivist obtain a comprehensive assessment of the hemodynamic profile using echocardiography.

## 1. Introduction

Invasive hemodynamic disturbances are common in intensive care medicine. During the past four decades, monitoring has evolved from simple manual blood-pressure measurement, to continuous invasive arterial blood-pressure monitoring, to thermodilution techniques using transpulmonary thermodilution (PICCO^®^) or invasive pulmonary artery catheter (Swan–Ganz catheter). These tools are often used in cardiac critical care due to the comprehensive evaluation of cardiac and circulatory function. In particular, pulmonary artery catheters carry a high inherent risk of complications, some of which are fatal. They also lose diagnostic detail in settings where only parts of the myocardium are affected (e.g., in regional wall-motion abnormalities during acute myocardial infarction or a regional pericardial tamponade). Changes will only be shown when they affect overall cardiac function. In daily practice, most of the parameters of the Swan–Ganz catheter can also easily be assessed less invasively with the 21st relative of the Swan–Ganz catheter: echocardiography.

Echocardiography as a diagnostic tool clearly impacts patient outcomes in life-threatening conditions (e.g., new wall-motion abnormalities due to myocardial infarction). However, even in less dramatic situations, echocardiography can provide the same information as advanced hemodynamic monitoring, generating beneficial effects for patient management. This is clearly seen in scenes wherein echocardiography has become part of the mainstay of therapy as it is in adult cardiac surgery, where it improves perioperative outcomes [1]. Beyond the cardiac surgical theater, echocardiography can provide crucial diagnostic information for critical care physicians. For example, in the context of hemodynamic instability, a focused echocardiographic examination can provide important clues about its cause and related therapeutic options [2]—before surgery, intraoperatively and after the operation, and without the need for instrumentation (as advanced hemodynamic monitoring would require). Similarly, echocardiography guides the use of volume resuscitation, vasopressor need, or inotropic therapy in critically ill cardiac and noncardiac surgical patients, as well as advanced hemodynamic monitoring. Furthermore, simple protocol-based examinations identify life-threatening clinical conditions such as severe heart failure or high-grade aortic valve stenosis [3]. Theoretically, the objective of every echocardiographic examination is to obtain a complete picture of cardiac function. This requires the echocardiographer to actively shift focus to areas such as a separate evaluation of valve and ventricular function, right and left ventricles, atria, systolic and diastolic function, preload and afterload, pericardium, and adjacent vessels. Individual findings are integrated into an overall picture and allow an assessment of hemodynamic status that supports informed clinical decisions. This will lead to a comprehensive judgment of the hemodynamic status. Here, we will review how echocardiography can serve as a tool to provide a bedside evaluation of cardiac function in the cardiac critical care setting.

## 2. Basic Modes of Image Acquisition

Ultrasound-based cardiac imaging has transformed clinical care in intensive care medicine, as well as in the operating room, and today forms an important column of comprehensive cardiac care. Theoretically, ultrasound machines use two different modes of image acquisition: M- and B-modes. M-mode is the oldest mode and records images along a sound axis over time. The B-mode represents the classical representation of tissue-specific sound conduction properties in a two-dimensional (2D) image. Both techniques are regularly used to characterize both right ventricular (RV) and left ventricular (LV) functions. 

In addition to image acquisition, blood flow and tissue velocity can be visualized and quantified using two different modes of Doppler echocardiography: pulsed-wave (PW) and continuous-wave (CW) Doppler. Both record flow velocities over time but with intrinsic differences between the two techniques. The PW Doppler mode can record flow velocities with high spatial resolution in a defined range but fails in the setting of higher velocities (up to ~150–170 cm/s). CW Doppler can resolve high-flow velocities but lacks spatial resolution. To assess flow velocity in relation to anatomical structures, one relies on color Doppler. Here, different colors are coded for the flow velocities depending on the direction of the blood flow and the velocity. Typically, red is used for flow directed towards the ultrasound probe, and blue is used for flow directed away from the ultrasound probe. 

All image acquisition can be applied as transthoracic echocardiography (TTE) or transesophageal echocardiography (TEE). TTE offers the intrinsic advantage of being noninvasive and rapidly available, and most guidelines, reference values, and scientific evidence refer to data from studies with TTE. Unfortunately, TTE requires access to the thorax, an anatomical site that is not easily accessible during surgery and can be challenging in patients after cardiothoracic surgery or under mechanical ventilation. 

Therefore, in the perioperative setting, physicians are more commonly relying on TEE, which commonly provides excellent high-resolution image quality due to proximity of the ultrasound probe to cardiac structures. Currently, mainly multiplanar TEE probes are used, allowing all degrees of freedom (flexion, rotation, translation) for image acquisition. Ultrasound devices and probes have progressed steadily, and the latest generations allow for more advanced measurements, using three-dimensional (3D) echocardiography and semiautomatic endocardial and myocardial tracking (so-called speckle-tracking echocardiography, STE). 

## 3. Assessment of Left Ventricular Function

Evaluation of systolic LV function is probably the most common investigation in perioperative echocardiography. There are numerous well-validated parameters to assess LV systolic function, which predicts perioperative morbidity and mortality [4,5,6]. Furthermore, diastolic relaxation also influences perioperative outcome [7].

Left ventricular function can now to a large extent be assessed with non-invasive echocardiography measurements, often replacing the more invasive technique such as left heart catheter. Modern echocardiographic machines have integrated various complex echocardiographic assessments into their software, but they still require a high level of training and are typically time-consuming. However, time is a precious resource for the acutely deteriorating patient in the ICU. Therefore, clinicians frequently rely on eyeballing techniques in the decision-making process. For the inexperienced user in a time restraint setting, eyeballing techniques can be “supercharged” using basic imaging modalities in echocardiography. The following paragraphs show several easy-to-learn parameters that can help to quickly assess the left ventricular function without using complex measurement tools.

### 3.1. Systolic LV Function

Systolic left ventricular function is an integral part of the point-of-care assessment of hemodynamically unstable patients. Some views (in TTE or TEE) help to confirm or exclude numerous pathologies as the cause of clinical deterioration. However, all examinations, regardless of the underlying imperative, should include a statement about the systolic LV function, which represents the approach recommended by International Guidelines [8].

Especially in emergencies, the investigator should not spend too much time on differentiated diagnostic algorithms but focus on the elimination of potentially life-threatening conditions. For this reason, most of the following techniques allow even the inexperienced examiner to quickly obtain information on left ventricular systolic function by “eyeballing.” 

#### 3.1.1. Dimensions and M-Mode Function Parameters

As a first step in the examination, it is recommended to obtain an overview of the diameters and wall thicknesses of the left ventricle. The M-mode offers the best temporal resolution of all ultrasound modalities with a sampling rate of at least 1000 frames per second, making fast or subtle movements optimally visible. Furthermore, the rather abstract image of the M-mode contains less ‘optical noise’ than a B-mode image for the inexperienced examiner.

##### Fractional Shortening

**Figure 1:**
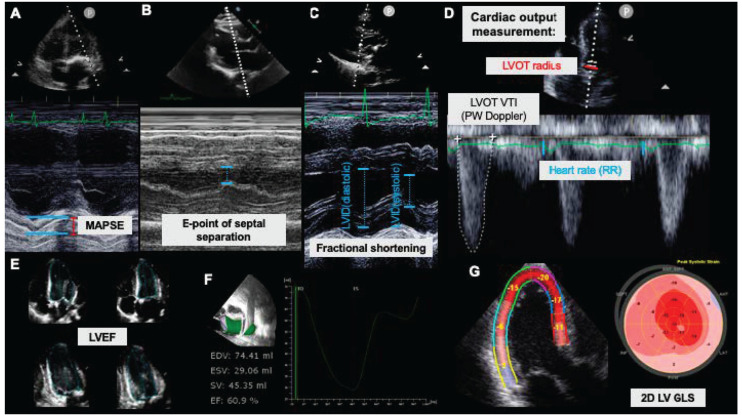
Echocardiographic evaluation of left ventricular systolic function. (**A**) Measurement of mitral annular plane systolic excursion (MAPSE, mm) using M-mode in transthoracic apical four-chamber view. (**B**) Quantification of the E-point of septal separation during early diastolic inflow in the transthoracic parasternal long-axis plane. (**C**) Fractional shortening is measured in the long-axis view and reflects the systolic circumferential function of the left ventricle. (**D**) By measuring the velocity time integral (VTI) with the PW Doppler in the left ventricular outflow tract (LVOT) while including the LVOT radius and heart rate (RR interval) into the calculations, the cardiac output can be quantified noninvasively. (**E**) The Simpson method derives LV volumes and left ventricular ejection fraction (LVEF) from four- and two-chamber views. This requires tracing of the endocardial border in the end-diastolic and end-systolic frames. (**F**) Three-dimensional echocardiographic acquisition of the entire left ventricle provides accurate volumetry and LVEF measurement. The technique is usually combined with semi-automatic speckle-tracking to allow for quick and robust analyses. (**G**) Speckle-tracking is further applied to 2D long-axis views of the left ventricle to measure longitudinal strain. The 2D LV global longitudinal strain of the LV (GLS) is a parameter with a better prognostic power compared to LVEF. EDV: end-diastolic volume; ESV: end-systolic volume; LVID: left ventricular inner diameter; SV: stroke volume.

To eject blood into the aorta, the left ventricle must also contract circumferentially. This change in diameter is described by fractional shortening (FS) and expresses the change in the diameter of the LV in M-mode from diastole to systole as a percentage (Figure 1C). Calculating FS requires a visualization of the plane that represents the maximum (end-diastolic) and subsequent minimum (end-systolic) dimensions of LV during a cardiac cycle. In TTE, the parasternal long axis view just below the level of the mitral valve tips is used. A suitable TEE view is the short mid-papillary transgastric axis. Calculation according to the formula below is performed automatically using modern echocardiography equipment:

$$\mathrm{Fractional}\;\mathrm{Shortening}\;\left(\%\right)=\frac{\mathrm{LVID}\left(\mathrm{diastolic}\right)-\mathrm{LVID}\left(\mathrm{systolic}\right)}{\mathrm{LVID}\left(\mathrm{diastolic}\right)}\times 100$$

LVID = left ventricular internal diameter

An FS of <25% indicates systolic dysfunction of the LV. Values above the upper reference limit of 45% may indicate a hyperdynamic circulatory state.

##### Mitral Annular Plane Systolic Excursion

To eject blood volume into the systemic circulation, the LV describes a pronounced longitudinal shortening in the direction of the cardiac basis in addition to circumferential narrowing (which is described by the fractional shortening, see above). This longitudinal movement can be quantified diagnostically by determining the mitral annular plane systolic excursion (MAPSE). MAPSE is a simple and well-validated marker. It is derived from the M-mode of the summative longitudinal function of the LV and closely correlates with the global function of the LV (Figure 1A). Especially in emergencies, MAPSE may be easier to collect for untrained investigators than more complex measurements, because it is easier to acquire in the presence of limited image quality [9]. In TEE MAPSE can be assessed in the apical four chamber view; in TEE the midesophageal four chamber view can be used. For this purpose, the M-mode beam is placed through the annulus of the lateral mitral annulus in the apical four-chamber view. Its systolic displacement is then measured in millimeters from the lowest point (end-diastolic) to the highest point (end-systolic). A MAPSE of 10 mm indicates a preserved left ventricular ejection fraction, whereas a MAPSE of less than 8 mm is associated with a decreased left ventricular ejection fraction (LVEF). For the most accurate measurements, the ultrasound axis should be perpendicular to the blood–endocardium interface, as the axial resolution is more precise than the lateral resolution.

When mitral annulus calcifications are present, MAPSE should be interpreted with caution, because this condition might limit the movements of the mitral annulus and thus reduce MAPSE. Furthermore, wall abnormalities of the mitral valve annulus could also contribute to a reduced MAPSE. This is important to keep in mind when evaluating LV function, especially when values are different from other measurements or the overall look of ventricular function.

##### E-Point of Septal Separation

During early diastole, the mitral valve opens fully, and the leaflet of the anterior mitral valve moves toward the interventricular septum. This movement of the anterior mitral valve leaflet is only possible when the ejection of blood into the ascending aorta is intact. If the ejection fraction is reduced, the blood volume within the left ventricle will remain elevated at early diastole. This, in turn, will reduce the movement of the anterior mitral valve leaflet. This can be used diagnostically [10]. For this, the smallest distance between these two structures is called the E-point of septal separation (EPSS) (Figure 1B). As an eyeball technique, the focus should be on the anterior mitral valve leaflet and if it fully opens. In TTE, we can measure the EPSS in the parasternal long-axis view using the M-mode. For this purpose, the axis runs through the tip of the anterior mitral valve leaflet, and the distance between the valve leaflet and the septum is measured in millimeters. EPSS correlates very well with systolic LV function. An EPSS of >7 mm indicates impaired LV systolic function.

EPSS should be interpreted with caution in the setting of impaired mitral valve opening, as it occurs in mitral valve stenosis. Here, the ejection fraction may be intact, but the EPSS is reduced because the mitral valve cannot open fully due to limited motion of the anterior leaflet. 

#### 3.1.2. Cardiac Output Determination through Doppler Volume–Time Integral

There are situations in a clinical setting where patients deteriorate over time. Here, repetitive measurements of cardiac outcome can influence the clinical management and guide vasopressor or inotropic therapy (e.g., heart failure with reduced ejection fraction; hyperdynamic circulation in shock). 

Cardiac output (CO) determination by echocardiography is an important alternative to more invasive methods, such as pulmonary catheterization or transpulmonary thermodilution. To measure the stroke volume and therefore cardiac output, Doppler echocardiography or 2D measurements can be used.

Conceptually, the LV ejects blood into a cylinder (ascending aorta) with each contraction. The base of this cylinder is the systolic cross-sectional area of the left ventricular outflow tract (LVOT), while its height is the distance the blood travels during ejection for that beat. From this, the volume per beat can be calculated, reflecting stroke volume. First, the diameter of the LVOT is measured. Assuming that the base of the LVOT cylinder is circular, the radius can be used to calculate its area. However, care must be taken, as small errors in 2D diameter measurements will lead to large errors in calculating the cross-sectional area, as the radius (half the diameter) is squared in the calculations. The LVOT diameter can be measured in long-axis or three-chamber views. Thereafter, the bloody flow velocity in the LVOT is measured using the PW Doppler at roughly the same spot as the diameter measurement, and the velocity–time integral (VTI) is formed (Figure 1D). The stroke volume is then calculated using the following formula:

$$\mathrm{stroke}\;\mathrm{volume}\;\left(\mathrm{mL}\right)=\mathrm{LVOT}\;\mathrm{area}\;\times \mathrm{VTI}$$

Multiplying the stroke volume by the heart rate at the time of measurement yields CO (L/min). Normalized to the patient’s body surface area, the result is the so-called cardiac index (L/min/m^2^). Theoretically, stroke volume can also be determined using volumetric methods (see below).

#### 3.1.3. 2D and 3D Volumetry

There are different methods for determining LV volumes from 2D echocardiographic images, all based on geometric assumptions. The most widely used method in practice is the modified Simpson’s rule of discs, also known as the Simpson method. Changes in ventricular volume (the systolic volume and the diastolic volume) also give away the stroke volume of the ventricle, which can serve as an alternative method of cardiac output measurement (please see above). To determine LV volume, the sum of a series of parallel slices from the apex to the base is calculated electronically. The advantage of this technique is that, even in the presence of altered ventricular geometry (e.g., dilated cardiomyopathy), volume determination produces reliable values compared to cardiac magnetic resonance [11] imaging. The measurement is performed in an apical view (TTE) or a four-chamber midesophageal view (TEE). The analysis is supplemented by a second measurement in the corresponding two-chamber view. Measurements at different times in the cardiac cycle yield end-diastolic volume (LVEDV) and end-systolic volume (LVESV). The LVEF is then calculated using the following formula:

$$\mathrm{LVEF}\;\left(\%\right)=\frac{\mathrm{LVEDV}-\mathrm{LVESV}}{\mathrm{LVEDV}}\times 100$$

Although 2D volumetry usually provides results that correlate well with reference technologies, more accurate measurements are achieved by using 3D echocardiography [11]. For this purpose, the raw data are segmented with special user-friendly semi-automatic tracking techniques based on STE. This allows fast, robust, and reproducible detection of endocardial borders and tracking throughout the cardiac cycle.

#### 3.1.4. LV Strain

Despite being the cornerstone for the evaluation of left ventricular systolic function, 2D measurements of left ventricular ejection fraction have some inherent shortcomings, such as interobserver variability. Structural damage to the heart is only given away when an impairment of LVEF is involved. Tools to evaluate subclinical left ventricular function can close the diagnostic gap. Left ventricular deformation measurements, such as left ventricular strain measurements from STE, close this diagnostic window, and offer additional advantages over 2D measurements of the left ventricle. The 2D STE of the LV measures the radial and circumferential shortening of the LV, while global longitudinal strain measures lengthening, shortening, thickening, and rotation capacity of the myocardium. Thus, it provides a more thorough assessment of the function of the left ventricle with a deeper understanding of the disease states.

The strain describes the relative change in myocardial length from end-diastole to end-systole and is expressed as a percentage. Negative values represent shortening, while positive values describe elongation. In fact, its prognostic value appears to be incrementally superior to LVEF (Figure 1E–G) [12]. Within certain limits, global longitudinal strain (GLS) integrates information on systolic and diastolic LV function, which is another strength compared to determining LVEF [13]. Using the ultrasound equipment available today, the bedside analysis of LV strain is possible within a few minutes after recording the images necessary for the measurement (four-, two-, and three-chamber views or a 3D data set). It is difficult to compare intraoperative TEE LV strain data with TTE values obtained from patients who breathe spontaneously. In its own right, intraoperative GLS values from TEE are associated with adverse outcomes, and values less than −17 to −20% are considered unfavorable with respect to prolonged hospital stay, the appearance of low cardiac output syndrome (LCOS), or postoperative atrial fibrillation [14]. While GLS is a not the first echocardiographic assessment a novice user should learn, a global understanding of what the LVEF and GLS visualize should be known to everyone performing an echocardiography.

### 3.2. Practical Considerations

All the mentioned parameters can be used to assess the left ventricular systolic function. However, they differ in clinical use and in the situation when they should be used. On a practical ground, we subdivide the above parameters into practicability and use. The parameters presented here can be categorized into parameters that can be used for estimation or parameters that highly correlate or even substitute invasive gold-standard measurement techniques. ‘Estimation’ parameters include FS, MAPSE, and EPSS; more exact evaluation parameters include stroke volume measurement by VTI, 2D or 3D volume measures (and ejection fraction), or left ventricular strain [15]. 

Parameters such as FS, MAPSE, and EPSS provide the advantage that no advanced calculation package is necessary for their measurement. As such, they can be used in simple bedside ultrasound machines, as long as the appropriate ultrasound probe is present. Thus, from a clinical and practical approach, we can use these parameters in acute deteriorating patients, or in the emergency of patients in shock. Here, they can help us to rule in or rule out certain differential diagnosis of patients in shock. In rapidly deteriorating patients, speed trumps perfection, and this is where the advantage of these parameters resides. For example, in patients with shock of unclear origin, we can quickly assess if cardiac function is impaired (EPSS, MAPSE below the reference range), or if the patients are hypovolemic (FS with increased values).

More exact parameters come into play when a thorough assessment of cardiac function is required. This is the case in patients with underlying cardiac disease who undergo therapy for a separate clinical condition (e.g., patient with known cardiomyopathy, treated for pneumonia) or patients admitted for cardiac conditions. Here, 2D or 3D volumetry stroke volume measurement or left ventricular strain measurement provide detailed information on cardiac function, sometimes even with beat-to-beat resolution. To measure these parameters, we require ultrasound machines that provide state-of-the-art speckle tracking-based calculation packages to semi-automatically determine the individual values. Otherwise, the values need to be measured offline with separate software packages. 

In summary, all left ventricular systolic parameters provide information for the clinical environment, but they always come with a trade-off. Simple parameters lose detail but gain ‘practicability’; advanced parameters provide accuracy but might take longer to be determined.

### 3.3. Diastolic LV Function

Relaxation of the left ventricle during diastole is an active process. In an adenosine triphosphate (ATP)-dependent process, the muscle fibers within the cardiac myocytes relax, which decreases the left ventricular intracavitary pressure significantly. When cardiac myocytes are depleted from ATP, this relaxation process is impaired. Physiologically, this process begins in late systole (with aortic valve closure; begin of diastole) and ends with mitral valve closure (end of diastole) and is known as the isovolumic relaxation period. It creates a suction from blood from the pulmonary veins from the left atrium into the left ventricle, without an increase in left ventricular chamber pressures. In diastolic dysfunction, this process is impaired, leading to an increased in left ventricular filling pressures. The evaluation of this in the perioperative setting is important, since LV diastolic dysfunction (heart failure with preserved ejection fraction) has a severe impact on patient prognosis and has an impact on catecholamine therapy [16].

#### 3.3.1. Doppler Echocardiography of the Transmitral Inflow Profile

**Figure 2:**
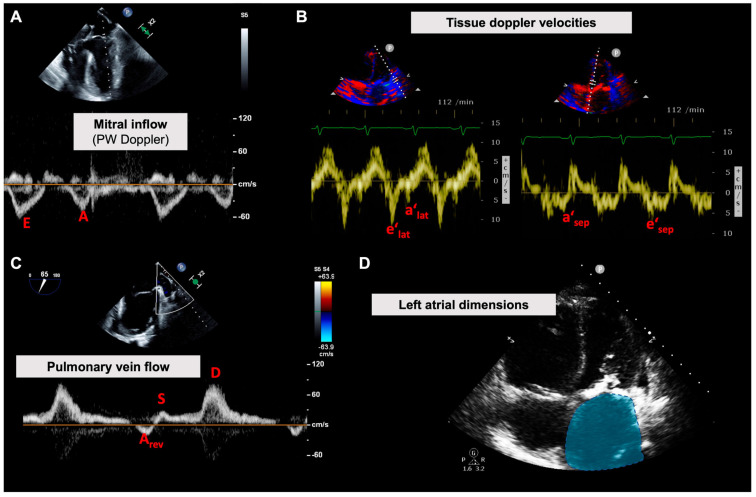
Echocardiographic evaluation of left ventricular diastolic function. (**A**) The blood flow velocities of the mitral valve inflow (E and A wave peak velocities) are measured using PW doppler; for example, in the four-chamber mid-esophageal view. (**B**) Using tissue doppler imaging, the corresponding tissue motion during diastolic inflow yields e‘ and a’ velocities, reflecting relaxation capacity and compliance of the left ventricle. (**C**) The pulmonary vein doppler flow profile can be acquired using both transthoracic and transesophageal echocardiography. While the A_rev_ flow is physiologically reversed, the reversal of the S and D flow can hint at increased left ventricular filling pressures. (**D**) The dimensions of the left atrium are easily acquired, for example here in the transthoracic apical four-chamber view. Enlargement of the left atrium is a sign of advanced diastolic dysfunction.

As mentioned above, the diastolic function (or dysfunction) is represented by the kinetics of ventricular filling. Therefore, it is important to look at ventricular filling dynamics when assessing the left ventricle works during diastole. The normal Doppler pattern of ventricular inflow is characterized by a short time interval between aortic valve closure and the onset of ventricular filling (isovolumetric relaxation time). After the opening of the mitral valve, there is a rapid inflow of blood from the left atrium into the ventricle. Blood flow velocities of 60 to 80 cm/s are achieved in healthy adults. This early maximum fill velocity (peak of the so-called E wave) occurs simultaneously with the peak pressure gradient between the atrium and the ventricle. After this peak velocity, the flow slows in healthy individuals. This time interval, defined as the deceleration time between the peak of the E wave and the intersection of a line following the deceleration slope with the baseline, is between 140 and 200 ms. Atrial contraction again causes an acceleration of flow through the mitral valve, which is expressed in the flow profile of the A wave, which reaches lower flow velocities (20 to 60 cm/s) under physiological conditions. In the case of impaired ventricular filling and increased filling pressures (diastolic dysfunction), atrial filling remains of greater importance, which is reflected in a decrease in the E/A ratio (normal E/A ratio 1–2) (Figure 2A).

#### 3.3.2. Tissue Doppler Echocardiography of the Mitral Valve Annulus

Due to ventricular filling during diastole, there is a geometric change in the left ventricle from the base to the apex. The velocity of this longitudinal myocardial lengthening can be measured by Doppler tissue imaging (TDI) and used for diagnostic purposes. The annulus of the lateral and septal mitral valves is used as a measurement site. Following Doppler measurement of blood flow velocities across the mitral valve, the TDI velocities of the mitral valve annulus are defined as e′ and a′, respectively (Figure 2B). The ratio of early transmitral blood flow velocity to TDI velocity (E/e′) may provide additional evidence of diastolic dysfunction.

Using the E/e′ ratio, the end diastolic pressure of the LV can be estimated non-invasively [17] as follows:

$$\mathrm{enddiastolic}\;\mathrm{LV}\;\mathrm{pressure}\;\left(\mathrm{mmHg}\right)=1.24\times \frac{E}{e^{\prime }}+1.9$$

For the diagnosis of diastolic function, all above parameters are usually used and evaluated using complex schemes. However, in the perioperative setting, a precise classification of the diastolic function is achieved using a simple algorithm [16]:

1. e′_lateral_ ≥ 10 cm/s: normal diastolic function
2. e′_lateral_ < 10 cm/s: diastolic dysfunction

E/e′ ≤ 8: diastolic dysfunction grade I (impaired relaxation)

E/e′ 9–12: diastolic dysfunction grade II (pseudonormalization)

E/e′ > 13: diastolic dysfunction grade III (restriction).

#### 3.3.3. Pulmonary Vein Flow Profile

The morphology, duration, and relative velocity of the pulmonary venous flow velocities are directly influenced by the left atrial pressure, as well as the compliance and contraction of the left ventricle. Therefore, the pulmonary venous flow profile indirectly reflects the dynamics of LV filling. Pulmonary venous flow velocities are measured by PW Doppler (Figure 2C). This is usually done in the right superior pulmonary vein in TTE and in the right or left superior pulmonary vein in TEE.

The flow profile is basically divided into two segments: the flow profile S during systole and D during diastole. In late diastole, even under physiological conditions, there is a slight reversal of flow back into the pulmonary veins, which is called A_rev_. If diastolic function decreases, left atrial pressure increases. As a result, the S wave of the systolic filling flattens, and the D wave increases proportionally. Due to increased resistance in the context of diastolic relaxation and disturbance of blood flow through the mitral valve during atrial contraction, the flow velocity of A_rev_ increases.

#### 3.3.4. Dimensions and Functional Analysis of the Left Atrium

Although the left atrium (LA) is easily characterized by echocardiography in dimensions and function, these measurements are rarely used perioperatively. An enlarged LA (2D: > 34 mL/m², 3D > 46 mL/m²) or LA dysfunction (LA strain ~<20%) both reflect advanced chronic diastolic LV dysfunction and are associated with increased end-diastolic LV pressures (Figure 2D). STE-based strain analysis is also increasingly used in LA function analysis and appears to be superior to LA volume determination alone (using 2D or 3D echocardiography) [18]. LA pathologies are strongly associated with the prognosis of most cardiac diseases, especially diastolic heart failure. On the contrary, nothing is known about the prognostic relevance of intraoperative LA analyzes.

##### Practical Considerations

Diastolic dysfunction has received less attention than systolic impairment in critically ill patients. However, the evaluation of diastolic function is essential in patients in the ICU whenever an echocardiography is performed. Diastolic dysfunction, in essence, leads to an increase in filling pressure, such as end-diastolic LV pressure (EDLVP). In the past, these measurements required invasive techniques, such as Swan–Ganz catheter placement, that carry substantial risks such as pulmonary artery rupture. Essentially, echocardiographic evaluation of EDLVP or wedge pressure estimation is less accurate but still provides integral information for patient management. 

The clinical scenarios where diastolic LV evaluation could be useful in the ICU could be cardiac deterioration, or post-surgical evaluation after valve surgery. In patients with a history of arterial hypertension, diastolic functional measurement can also help gauge the severity of impairment. In patients with pulmonary edema, the flow profile of the pulmonary vein provides additional information, because a blunted signal indicates incomplete decongestion of the pulmonary circulation (e.g., in acute pulmonary edema after acute systolic dysfunction). We regularly use a repetitive assessment of the pulmonary vein flow profile to guide the extent of diuretic therapy and fluid restriction. 

In summary, at least a brief evaluation of left ventricular diastolic function (e.g., by isolated quantification of e’lat) should be part of every echocardiography in the ICU, because it completes cardiac function in each individual patient and helps guide therapy.

## 4. Assessment of Right Ventricular Function

Since the RV differs from the LV in its complex three-dimensional structure and physiological properties, echocardiography poses a greater challenge for the inexperienced investigator. Due to its size and thin wall, the RV serves as a volume reservoir and therefore can tolerate preload increases (volume status, venous return) much better than afterload increases (e.g., in pulmonary artery embolism, hypoxic pulmonary vasoconstriction, or left heart failure). RV dysfunction can have many causes and is associated with a dramatic worsening of patient prognosis. The following sections will provide an overview of the possibilities of echocardiographic RV analysis.

### 4.1. Systolic RV Function

#### 4.1.1. Dimensions

**Figure 3:**
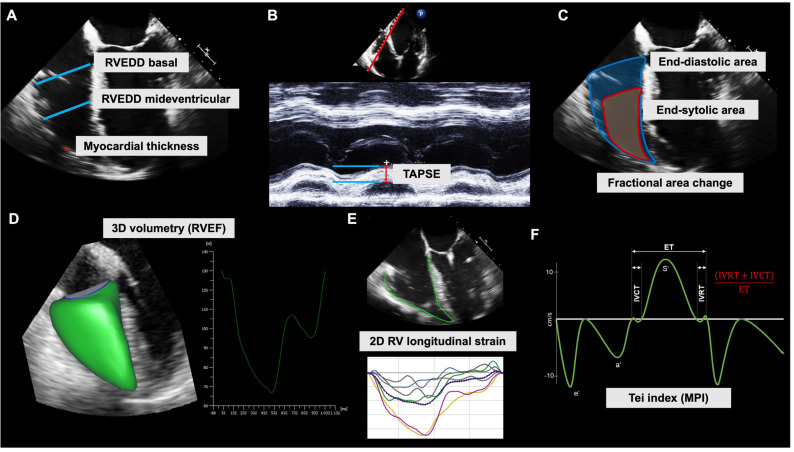
Analysis of right ventricular function. (**A**) Measurement of the enddiastolic diameter of the right ventricular (RVEDD) and the myocardial thickness of the free wall in the four-chamber mid-esophageal view provides a rapid analysis of the dimensions of the right ventricular. (**B**) Tricuspid annular plane systolic excursion (TAPSE, mm) using M-mode in apical four-chamber view reflects longitudinal right ventricular systolic function. (**C**) The relative change between the end-diastolic and end-systolic endocardial right ventricular area is called the fractional area change (FAC). FAC can be obtained in four-chamber transthoracic or transesophageal views and is a measure of systolic function. (**D**) Three-dimensional acquisition of the whole right ventricle and speckle-tracking-based analysis yields RV volumes, stroke volume, and ejection fraction (RVEF), avoiding geometrical assumptions and confounding 2D artifacts. (**E**) Strain analysis using speckle-tracking allows for the quantification of myocardial deformation, e.g., 2D RV longitudinal strain of the septal and free wall. (**F**) The Tei index (or myocardial performance index, MPI) is derived from isovolumetric relaxation time (IVRT), isovolumetric contraction time (IVCT), and ejection time (ET). This measure of global right ventricular function is usually calculated automatically after the manual definition of IVRT, IVCT, and ET in the tissue doppler profile of the basal right ventricular free wall.

To measure the dimensions of the right ventricle, a plane should be selected in which the RV is optimally visualized along its long axis and as circumferentially as possible. Transthoracically, this is typically achieved in the RV-focused four-chamber view, which in TEE can be approached from the mid-esophageal four-chamber view by slightly rotating the probe to the right side of the patient. Here, the end-diastolic diameter of the right ventricular (RVEDD) can be determined basally (near the mitral valve) and midventricular (at the level of the papillary muscles) (Figure 3A). Due to the complex geometry of the RV, RVEDD analysis is prone to error, especially in pathologically altered configurations. However, exceeding the reference range indicates RV dilation, which can be indicative of acute and chronic right heart failure (cor pulmonale). If RV pressures exceed LV pressures, a flattening of the interventricular septum can be found (so-called “D” sign). In chronic conditions, such as pulmonary hypertension, the thickness of the wall will increase. This can be best assessed by measuring the diameter of the compact myocardium (excluding the trabeculae) in the free wall region (Figure 3B). Planimetry (measuring the area within the endocardial boundaries excluding the trabeculae in end-diastole) allows for a somewhat easy way to analyze the RV configuration.

#### 4.1.2. 2D Function Parameters: TAPSE and FAC

In addition to dimensions, 2D echocardiography rapidly provides simple and robust parameters of RV function. The most important and still widely used parameter is the TAPSE (tricuspid annular plane systolic excursion). In its original form, it can only be obtained from the four-chamber transthoracic view using an M-mode sector through the lateral tricuspid annulus (Figure 3C). The TAPSE here indicates the absolute translation of the tricuspid annulus apically in millimeters and, strictly speaking, is a purely longitudinal functional parameter of the RV. However, it strongly correlates with global RV function and therefore with prognosis in the context of RV dysfunction. In TEE, a surrogate of TAPSE can be assessed in M-mode in the transgastric RV inflow plane (100° to 130°) or estimated by manual measurements in the B-scan of the four-chamber view, but these values cannot be directly compared with the TTE values. Recent approaches to derive a modified TAPSE from TTE using the anatomical M-mode (which generates M-mode recordings along any axis in regular B-images) have been promising [19]. The so-called FAC (fractional area change) refers to the systolic percentage change in the RV area and is therefore easily referred to as the “2D ejection fraction” of the RV (Figure 3B). Although most devices automatically output FAC after measuring EDA and ESA, it can also be quickly calculated manually using the following formula:

$$\mathrm{FAC}\;\left(\%\right)=\frac{\mathrm{EDA}-\mathrm{ESA}}{\mathrm{EDA}}\times 100$$

Values of <35% are considered pathological in patients awake and spontaneously breathing. In patients with severely abnormal RV configurations, the FAC can sometimes deviate significantly from the ‘true’ right ventricular ejection fraction (RVEF), usually due to ‘blind spots’ in the FAC (e.g., failure to account for the right ventricular outflow tract).

#### 4.1.3. 3D Volumetry and RVEF

The complex geometry of the RV can be fully visualized only using 3D imaging techniques. TEE is typically associated with optimal examination conditions (sedated/anesthetized, ventilated patients), allowing high-resolution 3D data sets to be generated using multi-beat recordings triggered by ECG (caution: atrial fibrillation, stitch artifacts). Therefore, 3D-based real-time methods make volumetry feasible at the bedside and provide a relevant prognostic RVEF in addition to right ventricular end-diastolic volume (RVEDV) and right ventricular end-systolic volume (RVESV) (Figure 3D–F). The method has been validated against the gold standard, cardiac magnetic resonance imaging, in many studies [20,21,22]. Although RVEF is one of the many parameters dependent on preload and afterload. Currently, there is evidence that the RV contraction pattern can change (longitudinal function, circumferential function), particularly in the context of major cardiac surgery, while RVEF remains relatively stable [23]. Because the end-diastolic volume of RV is larger than that of LV, the reference ranges for RVEF are correspondingly lower, because both ventricles eject the same cardiac output at identical heart rates.

#### 4.1.4. RV Strain

RV strain analysis was initially mainly experimental in nature and was often performed using LV strain software. Currently, there are many studies that highlight the prognostic importance of RV strain in various settings, including perioperative medicine [24,25,26,27,28,29,30]. Because the RV generates a crucial fraction of its volume ejection through longitudinal contraction, longitudinal strains are often regarded a meaningful correlate of global RV function. Despite growing evidence of an equivalent role of circumferential RV contraction, non-longitudinal RV strain measurements are difficult to derive from 2D recordings [31]. Using appropriate four-chamber view images focused on RVs from both TTE and TEE, a longitudinal 2D RV strain can be quickly and easily assessed using STE. In addition to the RV global longitudinal strain (normal < −23%), longitudinal free wall strains (normal < −27%) and septal wall strains (normal < −20%) are regularly distinguished [32]. In cardiac surgical patients, the determination of RV strain appears to be a better prognostic than the FAC measurement [24]. The question of which normal values apply in the anesthetized patient and what additional benefits 3D-RV strain analyzes have is the subject of current research [30].

##### Practical Considerations

The echocardiographic evaluation of right ventricular function has gained increasing interest in recent years, as fundamental evidence for the crucial prognostic impact of RV dysfunction in various cardiac and non-cardiac conditions exists. Analogously to the left ventricular function, parameters of the right ventricular function can be divided into parameters that are easy to measure at the bedside, but less predictive, and parameters that require off-line calculation packages.

Parameters, such as right ventricle dimensions, TAPSE, and RV FAC can be measured with most ultrasound machines with the appropriate probe and thus provide a rather quick assessment of the right ventricle. The parameters are useful in clinical situations where a quick assessment of the right ventricle is necessary, for example, in patients where a sudden increase in right ventricular afterload is suspected (e.g., pulmonary emboli, right ventricular dysfunction due to positive pressure ventilation).

For a full-scale assessment of right ventricular function, for example in progressive right heart failure, the more elaborate measurements such as 2D RV ejection fraction or RV strain can provide a complete picture of the patient situation. This might be helpful in patients after cardiac surgery or with chronic pulmonary hypertension. Today, 3D-based quantification of RV function has emerged as the echocardiographic gold standard, but its routine use is currently limited to a few centers with the necessary expertise and equipment.

In summary, all functional parameters of the right ventricle provide information for the clinical environment. In the acute situation, we recommend using fast and accessible functional parameters; when a thorough assessment is required to monitor therapy measurements for RV strain, it might provide a better picture of the clinical situation.

### 4.2. RV Doppler Echocardiography

#### 4.2.1. Estimation of Pulmonary Arterial Pressure Using CW Transtricuspid Doppler

**Figure 4:**
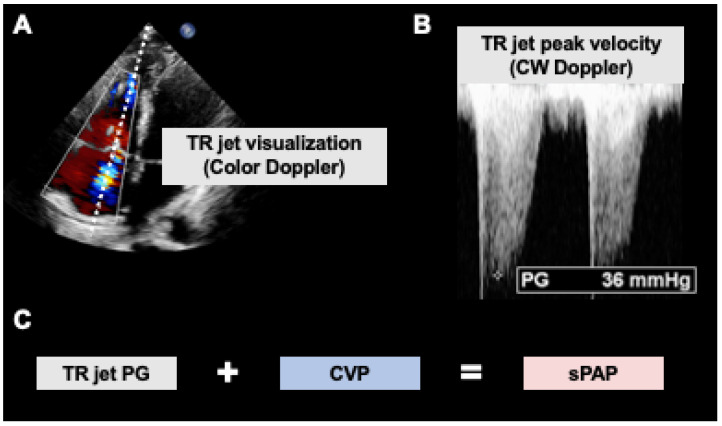
Noninvasive estimation of systolic pulmonary artery pressures using echocardiography. (**A**) Tricuspid regurgitation (TR) is visualized using the color Doppler mode in the four-chamber transthoracic view. (**B**) PW doppler recording of the TR jet yields its peak velocity during systole, which can be converted into a peak pressure (PG) by the modified Bernoulli equation (automatically). (**C**) The measured PG defines the difference in pressure between the right atrium and the right ventricle. Hence, central venous pressure (CVP) must be added to PG to derive the systolic pulmonary artery pressure (sPAP). The CVP can be read out invasively using a central line or estimated according to the collapsibility of the inferior vena cava.

Because acute and chronic elevations of afterload are often associated with RV dysfunction, determining systolic pulmonary artery pressure (sPAP) plays a critical role in initiating appropriate therapy. The invasive procedure of choice, right heart catheterization using Swan–Ganz catheters, is complex and can be associated with life-threatening complications. On the contrary, if RV dysfunction has been detected by echocardiography, a non-invasive Doppler-estimate can assess sPAP. Two important conditions must be met for measurement: (1) the presence of tricuspid regurgitation (TR) and (2) the CW Doppler beam must be parallel to the tricuspid insufficiency jet to avoid dramatic underestimation of sPAP. For this measurement, the TR is best visualized in the apical four-chamber view in TTE or in an atypical bi-caval view in TEE. According to the modified Bernoulli equation, echocardiography devices can automatically calculate a systolic pressure from the maximum flow velocity of the TR jet, which corresponds to the difference in pressure between RV and RA (Figure 4A,B). By adding the central venous pressure (measured or estimated; see below), we finally obtain the RV systolic pressure or sPAP, which is well-correlated with the invasively measured value [33].

#### 4.2.2. Tricuspid Valve Inflow Profile

PW Doppler can be used to record the inflow profile through the tricuspid valve in suitable sections (e.g., four-chamber view). This can provide valuable information on the diastolic function. Diastolic RV dysfunction is often a consequence of acute myocardial ischemia, in the context of volume challenge or increased pressure in the pulmonary circulation. Echocardiography can detect even mild deterioration of right heart failure. Determining the E/A can be easily acquired and helps to gauge the severity of impairment of right ventricular function.

#### 4.2.3. Tissue Doppler Echocardiography of the Tricuspid Annulus

The unique feature of TDI of the RV is that it provides parameters to evaluate both the systolic and diastolic functions of the RV. Doppler focus is placed on the lateral tricuspid annulus. Maximum systolic velocity (S′), as well as early (e′) and late (a′) diastolic tissue velocities, can be measured. Comparison of these parameters with reference values can be used to detect RV systolic or diastolic dysfunction. Furthermore, the so-called myocardial performance index (MPI or Tei index) can be used to determine a TDI-based measure of the global RV function. For this purpose, different times are measured from the recorded TDI course of a cardiac cycle, and the MPI is calculated (usually automatically).

#### 4.2.4. Diameter and Collapsibility of the Inferior Vena Cava

Echocardiographic evaluation of the inferior vena cava (VCI) provides valuable information on volume status and central venous pressure (CVP). For this purpose, the maximum diameter of the VCI is measured in a subxiphoid view, and the collapsibility during (active) inspiration is observed. A decrease in diameter with spontaneous collapsibility indicates an intravascular volume deficiency in the systemic venous circulation (e.g., in shock). On the contrary, increases in diameter (>2.1 cm) and reduced collapsibility (<50%) indicate an increase in CVP (10–20 mmHg). Although diastolic RV dysfunction can therefore be suspected, this method is suitable for using the estimated CVP to determine the sPAP as mentioned above. Intraoperatively, CVP estimation is obsolete, as invasive CVP measurement can be performed, and the collapse of the VCI does not produce meaningful results in ventilated patients.

##### Practical Considerations

Doppler assessment of RV should be performed in all patients undergoing echocardiography for hemodynamic deterioration because the measurements are crucial for macro dynamics. As such, Doppler sonographic measurements of tricuspid valve pressure gradient can be used in patients who undergo invasive mechanical ventilation for respiratory failure (such as acute respiratory distress syndrome). Here, the measure can provide an excellent estimate of the right ventricular after load of the right ventricle.

If hypovolemia such as that in acute hemorrhage is suspected, the assessment of the vena cava is helpful, because with a little training, even the novice can detect changes that might affect the patient’s macrohemodynamics.

In summary, Doppler measurements complete the assessment of the right ventricular function because they put functional measurements in context with hemodynamic parameters.

## 5. Conclusions

Echocardiographic measurements can substitute advanced hemodynamic measurements in many settings. It comes with the advantage that even TEE carries a substantially lower risk of complications than, e.g., Swan–Ganz catheter placement.

## Data Availability

Not applicable.
